# Women's position and attitudes towards female genital mutilation in Egypt: A secondary analysis of the Egypt demographic and health surveys, 1995-2014

**DOI:** 10.1186/s12889-015-2203-6

**Published:** 2015-09-10

**Authors:** Ronan Van Rossem, Dominique Meekers, Anastasia J. Gage

**Affiliations:** Department of Sociology, Universiteit Gent, Korte Meer 3-5, 9000 Ghent, Belgium; Department of Global Community Health and Behavioral Sciences, Tulane University, School of Public Health and Tropical Medicine, 1440 Canal Street, New Orleans, LA 70112 USA

## Abstract

**Background:**

Female genital mutilation (FGM) is still widespread in Egyptian society. It is strongly entrenched in local tradition and culture and has a strong link to the position of women. To eradicate the practice a major attitudinal change is a required for which an improvement in the social position of women is a prerequisite. This study examines the relationship between Egyptian women’s social positions and their attitudes towards FGM, and investigates whether the spread of anti-FGM attitudes is related to the observed improvements in the position of women over time.

**Methods:**

Changes in attitudes towards FGM are tracked using data from the *Egypt Demographic and Health Surveys* from 1995 to 2014. Multilevel logistic regressions are used to estimate 1) the effects of indicators of a woman’s social position on her attitude towards FGM, and 2) whether these effects change over time.

**Results:**

Literate, better educated and employed women are more likely to oppose FGM. Initially growing opposition to FGM was related to the expansion of women’s education, but lately opposition to FGM also seems to have spread to other segments of Egyptian society.

**Conclusions:**

The improvement of women’s social position has certainly contributed to the spread of anti-FGM attitudes in Egyptian society. Better educated and less traditional women were at the heart of this change, and formed the basis from where anti-FGM sentiment has spread over wider segments of Egyptian society.

## Background

Female genital mutilation (FGM) is still a common practice in many African countries. The *World Health Organization* (WHO) estimates that worldwide between 100 and 140 million women have been cut [1], of which about 91.5 million in Africa. They also estimate that in Africa about three million girls are circumcised every year.

In Egypt, FGM remains nearly universal: over 95 % of women between 15 and 49 years old are circumcised, and this proportion remains fairly constant across all cohorts [2 and own calculations]. WHO distinguishes four types of FGM [3]. In Egypt types I (clitoridectomy) and II (clitoridectomy + (partial) removal of the labia minora) are the most frequent ones [4]. Type III (infibulations) is fairly rare, as is type IV (other forms). The practice usually takes place before puberty [5]. The median age at circumcision is 10 years of age, and almost all girls are cut before their 13^th^ birthday [2]. Traditionally, the cutting was done by Dayas, traditional midwives, but the practice is increasingly medicalized [5, 6]. By 2005, more than 70 % of the cuttings were performed by doctors and only 22 % by Dayas [2].

For over half a century Egypt has been developing, to little effect, policies to discourage and ban FGM. An important reason for their failure is that FGM still enjoys the support of a large majority of the population [7, 8]. In 2003, only 23.3 % of ever-married women favoured its discontinuation [2], and 60.8 % believed that FGM is required by their religion. Although some prominent Islamic leaders have recognized that Islam does not require women to be cut [9] and even though the country’s supreme Islamic authorities reiterated it was prohibited [10, 11], many Islamic leaders still accept or even support the practice [4, 12, 13]. In 2007 and 2008 laws were passed that banned the practice [14, 15]. However, it remains unclear how rigidly these laws have been enforced. Although the 2007 law prohibited general practitioners from performing FGM, Rasheed, Abd-Ellah and Yousef [16] found that in Upper Egypt, the incidence of FGM remained very high, and that most cuttings were still performed by general practitioners. The social and political upheaval leading to and following the fall of the Mubarak regime in 2011 may further have undermined the enforcement of the laws as the Muslim Brotherhood is said to support the practice.

Although female genital mutilation or female circumcision is still nearly universal in Egypt, there is some evidence that the social and political climate regarding FGM is changing. According to the 2014 *Egypt Demographic and Health Survey* (EDHS) 92 % of ever married women between the ages of 15 and 49 were circumcised [17]. However, the prevalence of FGM among 20–24 year old ever married women was only 87 %, compared to about 95 % for 35 to 49 year olds. El-Gibaly et al. [6] also demonstrated that the prevalence of FGM among girls aged 10–19 was about 10 % lower than among their mothers. Other studies confirm these results [5, 18], suggesting a slow decline of the practice. Given the embedment of FGM in tradition and social structure and the widespread support for it, the eradication of this practice – which is the objective of current legislation -- seems impossible without major changes in popular attitudes. Theories of behaviour change stress the importance of attitudinal change as a necessary, although not sufficient, precursor to behavioural change [19–24]. For people to abandon traditional behaviours, such behaviours must be delegitimized while alternative ones need to gain acceptance.

The starting point of this study is that FGM is associated with the social position of women, i.e., their location in recognized status and role structures, and that the practice is culturally embedded and therefore widely supported. The delegitimization of the practice and an attitudinal change among large parts of society are essential steps in the abolishment of the practice. Countries where FGM is prevalent typically have high gender inequality. International organizations emphasize female empowerment, improving women’s position in society, and reducing gender inequality as a strategy to eradicate FGM [25] This paper examines the hypothesis that anti-FGM attitudes initially emerge among the more ‘modernized’ segments of Egyptian society, where women are believed to be more empowered, and subsequently spread from there to the rest of society.

In communities that practice FGM, people often accept it as a normal part of growing up as a woman. In traditional societies being cut confers status on a woman because it identifies her as a member-in-good-standing of her community. Parents fear that non-circumcised daughters will do less well on the marriage market than circumcised women. Specifically, parents worry that they will have more difficulty finding a spouse or will have to be satisfied with a lower status one. Whether a woman has been circumcised determines not only the moral standing of a woman, but also her social identity and status within the community [12, 26–30]. The practice of FGM stresses not only the subservient position of women in society, but also symbolizes girls’ coming of age and confirms them as full members of the community [4, 30]. Noncircumcised women risk being treated as outcasts, as immoral women. Not being cut leads to stigmatization and loss of status, for both the woman and her family [27, 31, 32]. Parents experience considerable social pressure to have their daughters cut [33, 34]. The social costs of not having one’s daughter cut can be quite substantial: loss of status, lower marriage opportunities for their daughters, social exclusion, etc. Several studies have shown that the non-circumcision of a daughter may lead to a loss of status and stigmatization, not just for the daughter herself, but for the entire family [13, 27, 31, 32, 35–38]. As FGM is linked to the position of women in society [12, 25, 28, 30], substantial social pressures exist to conform to the norm that states that FGM is a normal aspect of every woman’s life. The practice is strongly embedded in the society’s traditions and contributes to the social status of both the women themselves, and their families. The extent to which individuals and families can withstand such pressures and go against tradition depends on the available sources of status as well as on their exposure to other social environments and influences. Therefore, not all groups are equally likely to change their attitudes toward FGM.

The strong cultural embeddedness of the practice makes a shift in societal attitudes essential for a fundamental and long-lasting behaviour change and the final eradication of FGM. Most individual-level theories of behaviour change inspired either by social cognitive or rational choice theories recognize the role played by attitudes [19–22]. Attitudes reflect the relative values of both old and new behaviours, and might signify the readiness to change, although it does they may not necessarily lead to behaviour change. The problem with cognitive models of behavioural change is that they ignore the context in which decisions are made and assume that when an individual is convinced of the benefits of behavioural change he or she is also empowered to implement this change [39, 40]. Coale’s famous Ready, Willing, Able model [23, 24], already pointed out that one not only needs to be ready to change, but also willing and able to do so. According to this model all three conditions need to be met before an actual behaviour change occurs. As FGM is deeply entrenched in social traditions and cultural frames and is strongly connected with family social status simply being ready to stop the practice (as reflected by anti-FGM attitudes) usually will not be sufficient to trigger behaviour changes, as actors may neither be willing (due to the social costs of not cutting one’s daughters) nor able to do so (because of lack of power in the decision process). Nevertheless, an attitudinal shift remains essential. Although anti-FGM law enforcement can force people to change their behaviours, without attitudinal change, such changes in behaviours tend to be short-lived.

Attitudes are anchored in community structures and reflect one’s position within this community. Attitudes relating to gender, including FGM, therefore are linked with the position of women in the community. The stronger their position, the more likely they will be able to adopt a more ‘modern’ or ‘western’ view on these issues. The empowerment of women is often linked to processes of modernization: urbanization, increased education, industrialization, rationalization, individualization, emotional nuclearization, etc. [33, 38, 41–43]. Education is the key factor here, spreading modernity across society. Jejeebhoy [44] lists five ways in which education empowers women and thus may affect their reproductive health decisions and behaviours. First, by exposing women to outside influences education expands women’s knowledge beyond the common knowledge of the community and changes their outlook and values. Second, educated women are not only more confident, but also are in a stronger position relative to other family members with less education, which enhances their decision autonomy. Third, educated women tend to be more mobile and are thus better able to interact with actors outside the community. Fourth, education leads to a shift in loyalty away from the extended family and community and towards the nuclear family, which in turn leads to emotional nucleation. Finally and potentially most importantly educated women tend to have more control over material resources, largely because they tend to be more active in the money economy. In combination with employment in modern sectors of the economy, education provides women with alternative routes of status attainment. Marriage no longer is the only way for women to obtain status, and thus the social ‘need’ to have one’s daughters cut is lessened. Women with higher levels of autonomy are typically more likely to oppose FGM. For instance, Allam et al. [9] found that among the most modernized group in Egypt, i.e., university students, support for FGM was substantially lower than in the rest of society. Only 28 % of the students supported it. Those favouring its abolishment also had better knowledge of the dangers of the practice and tended to claim it had no advantages. Other studies found that mother’s education affects the likelihood that their daughter are cut or that they intend to have them cut [6, 45] or to oppose FGM [5, 6, 33, 46]. More emancipated women with greater autonomy tend to be guided less by tradition and less subjected to social control, and to also have better knowledge of the benefits and costs of FGM. For them and their daughters, marriage no longer is the only way to obtain status as they possess alternative routes of status attainment.

UNDPs *Gender Inequality Index* shows that gender inequality in Egypt declined slightly between 1995 and 2013. However, Egypt continues to have relatively high gender inequality, ranking 25^th^ (out of 130) in 1995 and 23^rd^ (out of 152) in 2013, indicating that progress in gender equality has been slower in Egypt than in most other countries [47]. El-Safty [48] reports that there has recently been a conservative backlash which is leading to a de facto curtailment of women’s rights. However, women’s progress has not been equal across all domains. For instance, Egyptian women have made considerable progress in terms of education, but much less in terms of labour force participation and employment. Female literacy among women age 15 and older increased from 22.4 % in 1976 to 65.8 % in 2012. Gross secondary school enrolment of women has increased from 20.4 % in 1970 to 87.8 % in 2013 [49]. However, the percentage of women aged 15 and older who are employed did not increase between 1990 and 2013. Although the employment rate of this age category fluctuates during this period, in 1990 26.7 % of women aged 15 and over were employed, and in 2013 only 22.9 % [49]. For many women modernization is only partial. Their education level might increase but they remain economically dependent on their husbands or families, limiting their autonomy. As this affects their position in society it is likely to influence their view on the role of women in Egyptian society.

In the modernization model the link between the change of one’s social position, one’s attitudes and behavioural change is quite straightforward. In reality the link may be less clear. Not all modernization processes run synchronously and women’s empowerment may be limited. Usually changes in attitudes are insufficient to trigger behaviour change, because decisions are rarely made in isolation from one’s social environment (family, community, etc.). Even for more individualistic decisions, such as whether to stop smoking or drinking, whether to use a condom or to change jobs, one needs to take into account one’s environment, and that may influence the decision. This certainly will be the case for FGM as this may affect a family’s social status. Therefore, the decision process is likely to be much more complex, involving not only the mother but also other family members. Because the family constitutes the primary unit of status, decisions pertaining to this status tend to be family affairs. The extent that women weigh in on these decisions, including decisions concerning FGM, depends on their position within the family and community. Often mothers have little control over the decision whether their daughters will be cut. They may oppose having their daughters cut, but neither be willing nor able to influence this decision.

The spread of new attitudes through a society is fundamentally a social process in which actors influence each other. New attitudes originate in specific subpopulations and then spread to the rest of society. Some of these (groups of) early adopters may serve as examples, role models, opinion leaders or reference groups for other segments of society. Innovation studies have demonstrated that changes are more likely to be initiated by small groups of innovators. Innovators have a “willingness-to-change” that makes them sensitive and receptive to new ideas and practices [50] and that leads them to engage in more cosmopolitan social relationships [51, 52]. Innovators often adopt such practices after exposure to external influences, through mass media etc. Research on the diffusion of practices in developing nations shows that access and exposure to these external resources is dominated by economically advantaged and less culturally traditional groups. Less advantaged and more traditional segments of society are not only less exposed to sources of innovation but are also ill positioned to take advantage of them. A number of studies have found that highly educated and urban women were more likely to favour the discontinuation of FGM [44, 53], and that wealthier women and better educated women were less likely to intend to have their daughters cut [45, 54]. Given the higher status of these groups they may serve as role models for other segments of society. Several theoretical models, including social cognitive theory [20], social convention theory [55], and diffusion of innovation theory [51, 56], note the importance of role models for behaviour changes. Role models can affect knowledge, attitudes, and behaviour through direct contact, but also through their visibility in the community and their status [51]. In most cases, such role models need to be well perceived by their audience. Marginal groups lack the status to fulfil this role. To the extent that the more modernized segments of Egyptian society serve as role models or reference groups for more traditional segments, anti-FGM attitudes are likely to spread from the former to the latter.

## Methods

### Sample

For this study we analysed secondary data from six waves of the *Egypt Demographic and Health Survey* (EDHS): 1995, 2000, 2003 2005, 2008, and 2014. The EDHS questionnaires focus on topics such as fertility, contraceptive use, infant and child mortality, maternal and child health, immunization, nutrition, as well as health related knowledge and attitudes. The surveys also included questions about FGM. Because the Demographic and Health Surveys is a series of standardized surveys, identical procedures were used for both the sampling and data collection in all EDHS waves. The EDHS surveys provide information about large nationally representative samples of ever married women. The only exceptions are the 2003 EDHS, which was a smaller interim DHS in which the Frontier Governorates were excluded from the sample, and the 2014 EDHS where two of the five Frontier Governorates were excluded because of security concerns. As less than 2%of the Egyptian population lives in those governorates, the impact on the representativeness of this survey is limited, and in the multivariate analyses we statistically controlled for region of residence. These six datasets contain information on a total of 97,274 ever-married women between the ages of 15 and 49 years. A three-stage sampling design was used. In the first stage, primary sampling units (shiakhas/towns and villages) were selected with probability of selection proportional to size. In the second stage, the primary sampling units were divided into parts of roughly equal size, and depending on the size of the primary sampling unit, between one and three parts were systematically. The selected parts were subsequently divided into smaller segments. Two segments were selected from PSUs that had two or three parts; one segment from all other PSUs. Within each selected segment, a systematic random of households was selected using a household listing. All ever-married women in the household between 15 and 49 years who were present in the household the night before were eligible to participate in the survey. More detailed information on the sampling and data collection is available in the EDHS reports [2, 17, 57–60]. The procedures and questionnaires of all DHS surveys have been reviewed and approved by the *ICF International* institutional review board (IRB) and comply with the *U.S. Department of Health and Human Services* regulations for the protection of human subjects (45 CFR 46), as well as by an Egyptian IRB to assure compliance with Egyptian rules and laws [61]. The de-identified EDHS data are publicly available.

### Variables

#### Attitude towards FGM

The dependent variable in this study indicates whether or not the respondents favour the discontinuation of FGM. The phrasing of the question in the various EDHS surveys varied only little, and not in a way it would affect the response. Only respondents who explicitly stated that they favoured the discontinuation of FGM were coded as opposing FGM, all other respondents (in favour of continuation, depends, other or don’t know) were coded as not opposing the practice. This approach provides a conservative estimate of the number of respondents favouring the discontinuation of FGM.

#### Woman’s social position

The social position of the respondent was measured by her education level (no formal education, primary, secondary, or higher education), literacy level (illiterate, partly literate, fully literate), her occupation, and the household’s possession of basic assets and amenities. As a proxy for household wealth, we included an index of basic assets and amenities. This index is defined as an unweighted count of up to eight items that the respondent’s household may possess (drinking water in residence, flush toilet, finished floor, electricity, radio, television, refrigerator and bicycle). The index was limited to these items because the index needed to be comparable over the six waves.^1^ Because of the items included and the way the questions were asked, this index only discriminates well among respondents at the lower end of the wealth distribution.

#### Control variables

As FGM is deeply entrenched in social, cultural and religious traditions, three variables were added that capture these traditions: whether the respondent herself is cut, her religion, and the number of children she has given birth to. Religious affiliation was coded as Muslim, Christian, or other/missing. The 2000 and 2003 surveys did not include a question on religion. Hence, information on religion is coded as missing for all respondents for those two survey years. Finally, the current age of the respondent, and the region and the degree of urbanization where the respondent resides were included. The urbanization variable distinguished between the capital and large cities, (smaller) cities, towns and the countryside. The 2014 EDHS only distinguished between rural and urban places of residence. For the purpose of the analysis, therefore, a dichotomous indicator for rural residence (0: urban, 1: rural) is used.

### Statistical analyses

Bivariate analyses using *χ*^2^ and ANOVA tests are used to analyse trends in both focal and control variables across the EDHS waves. These analyses test whether these variables changed in the population of ever-married Egyptian women. For categorical focal variables (education, labour market participation, and literacy) we subsequently used ANOVAs to test whether there are significant zero-order effects of these variables on the dependent variable. For continuous focal variable (household wealth), we tested this using bi-serial correlations.

To test whether the effects of the woman’s social position variables are robust, multilevel logistic regressions were run with opposition to FGM as the dependent variable. The first level in this analysis is formed by the individual women, the second by the EDHS wave. In a first model a random intercept model was estimated including all level 1 variables. In the second model, the EDHS wave (survey year) was added as a level 2 variable. For each level 1 variable, we subsequently checked whether its effect varied significantly across the EDHS survey waves. For six variables a significant random slope was observed: region, circumcision status, age, rural residence, education and labour market position. Separate analyses were run with an interaction term between each of these variables and the EDHS survey wave. The results for interaction terms that included the woman’s social position variables (education and labour market position) are reported in Figs. 3 & 4.

## Results

### Descriptive statistics

Table 1 shows the descriptive statistics for the pooled data. Opposition to FGM still remains a minority opinion. For the six EDHS waves combined only 22.5 % of the respondents favoured the discontinuation of FGM. A large proportion of Egyptian women are still in a fairly weak social position. A large minority of the women in the six EDHS waves had no formal education (34.9 %), while 39.0 % had secondary education, and a further 10.3 % higher education. Many women report to be illiterate (40.4 %), while a small majority (52.6 %) claim to be fully literate. This suggests a clear division in society regarding the education of women. As shown in Table 1 the overwhelming majority (81.1 %) of women in the pooled dataset are not working. Of those who were working 39.4 % held a professional, technical or managerial function, while another 23.1 % were working in clerical or service occupations, and 21.1 % in agriculture. Most respondents (51.9 %) live in households were (almost) all basic amenities were present, only 12.9 % of them lived in a household where only few of the basic amenities were present. On the average, a respondent possessed 6.02 out of 8 basic amenities and assets.

**Table 1:** Descriptive statistics for control variables

Variable	F	f
Should FGM be discontinued		
No	75310	77.5 %
Yes	21914	22.5 %
Region		
Urban Governorates	16967	17.4 %
Lower Egypt - Urban	11410	11.7 %
Lower Egypt - Rural	32421	33.3 %
Upper Egypt - Urban	11076	11.4 %
Upper Egypt - Rural	24417	25.1 %
Frontier Governorate	983	1.0 %
De facto place of residence		
Urban	40053	41.2 %
Capital, large city	16807	17.3 %
Small city	11717	12.0 %
Town	3905	4.0 %
Urban (2014)	7623	7.8 %
Rural	57221	58.8 %
Current age		
Mean & SD	33.13	8.65
15–19	3818	3.9 %
20–24	14359	14.8 %
25–29	19286	19.8 %
30–34	16721	17.2 %
35–39	16107	16.6 %
40–44	13816	14.2 %
45–49	13166	13.5 %
Total number of children ever born		
Mean & SD	3.14	2.24
0	9051	9.3 %
1	13559	13.9 %
2	20115	20.7 %
3	19792	20.3 %
4	13277	13.6 %
5	8216	8.4 %
6	5124	5.3 %
7+	8141	8.4 %
Ever circumcised		
No	4328	4.5 %
Yes	92894	95.5 %
Religion		
Muslim	69049	71.0 %
Christian	3447	3.5 %
Other/Missing	24778	25.5 %
Labor market participation		
Not working	78862	81.1 %
Prof., Tech., Manag.	7260	7.5 %
Clerical	2760	2.8 %
Sales	1429	1.5 %
Agriculture-self employed	1508	1.6 %
Agriculture-employee	2382	2.4 %
Services	1498	1.5 %
Skilled manual	1094	1.1 %
Other	481	0.5 %
Basic amenities index		
Mean & SD	6.02	1.74
Few (0–3)	7987	8.2 %
Some (4–5)	13415	13.8 %
Most (6–8)	75875	78.0 %
Highest educational level		
No formal education	33924	34.9 %
Primary	15408	15.8 %
Secondary	37894	39.0 %
Higher	10049	10.3 %
Literacy		
Illiterate	39217	40.4 %
Partially literate	6878	7.1 %
Fully literate	51088	52.6 %

Legend: *F*: absolute frequency, *f*: relative frequency

That FGM is well embedded in Egyptian society and culture is demonstrated by the finding that almost all women in the surveys reported to be circumcised; only 4.5 % said they were not. On average, women had given birth to 3.14 children (SD = 2.24); 9.3 % of the women had not (yet) given birth, and 13.7 % had more than 5 children. As a consequence of the lack of information on religion in the 2000 and 2003 surveys, 25.5 % of the respondents were coded as missing (although the overwhelming majority of them is Muslim), 70.1 % reported to be Muslim, and 3.5 % Christian.

The majority of the respondents lived in the countryside (58.8 %). Most respondents lived either in rural Lower Egypt (33.3) or rural Upper Egypt (25.1 %). Only 17.4 % lived in the Urban Governorates and 1.0 % in the Frontier Governorates. The average age of the respondents was 33.1 years. Only 3.9 % were younger than 20 and 27.7 % were between ages 40 and 49.

### Trends in attitudes towards FGM

**Fig. 1:**
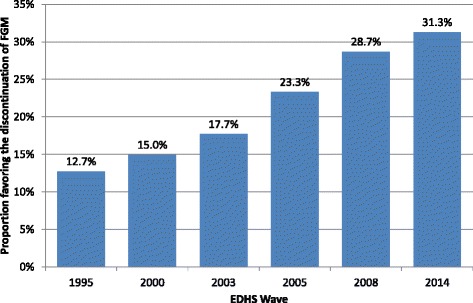
Trend in opposition to FGM among ever-married Egyptian women, 1995–2014

As shown in Fig. 1, opposition to FGM among ever-married women in Egypt has risen steadily from the mid-1990s onward (*χ*^2^(5) = 2767.2, p < 0.001). In 1995 only 12.7 % of ever-married women favoured the discontinuation of FGM; by 2014 this had more than doubled to 31.3 %. Figure 1 also shows that the increase in opposition to FGM occurred mainly from 2003 onward. Between 2003 and 2005 a larger increase in the proportion of women opposing FGM was observed than between 1995 and 2003. The increase slowed somewhat down in later years, especially between 2008 and 2014.

### Trends in the social position of women

**Fig. 2:**
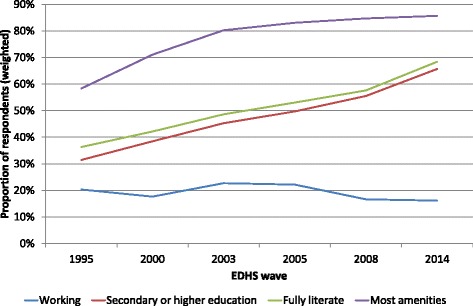
Trends in the education level, literacy, work status and basic amenities of eve-married Egyptian women, 1995–2014

Figure 2 illustrates the trends in ever-married women’s position between 1995 and 2014, as measured by their level of education, literacy, labour market participation and the household’s basic amenities index. The results show that women’s educational levels have risen sharply between 1995 and 2014 (*χ*^2^(15) = 5483.5, p < 0.001) . The percentage of women who had no formal education decreased from 43.7 % in 1995 to 24.0 % in 2014 (not shown), while the percentage of women with secondary or higher education increased from 31.5 % to 65.7 %. Literacy has also increased significantly (*χ*^2^(10) = 4718.2, p < 0.001), with the percentage who are fully literate increasing from 36.3 % in 1995 to 68.4 % in 2014.

Although the labour force participation of the ever married women in the EDHS varies significantly across the various EDHS waves (*χ*^2^(40) = 2479.6, p < 0.001), no clear trends can be distinguished. The proportion ever-married women who are employed remains a small minority fluctuating between 22.7 % in 2003 and 16.1 % in 2014. Most ever-married women in Egypt over the period studied are not working, and the data do not show any increase in female labour force participation over this period. The number of basic amenities possessed by the households also increased significantly between 1995 and 2003, then to remain fairly stable to 2008, and to decline again somewhat between 2008 and 2014 (*F*(5, 97,268) = 519.8, p < 0.001). Fig. 2 shows that the proportion with most or all basic amenities (scores ≥ 6) rose from 58.3 % in 1995 to 80.3 % in 2003 and to 85.7 % in 2014. The increase in proportion respondents who had most or all basic assets or amenities slowed down considerable over the past decade.

### Effects of women’s social position on attitudes towards FGM

All four indicators of a woman’s social position are significantly (at *p* < 0.001) associated with women’s belief that FGM should be discontinued. However, the relationships of education level (Ε^2^ = 12.1 %) and literacy (Ε^2^ = 8.7 %) with FGM opposition are substantially stronger than those of labour market participation (Ε^2^ = 3.5 %) and household wealth (Ε^2^ = 3.4 %). The belief that FGM should be discontinued increases significantly (*p* < 0.001) with each level of education. While only 8.9 % of the women with no formal education favoured the discontinuation of FGM, 13.2 % of the women with only primary education did so, and 29.7 % of those with secondary education. Among women with higher education, a majority (55.7 %) favoured the discontinuation of FGM. A similar relationship is observed with literacy. Only 8.8 % of the illiterate respondents favoured the discontinuation of FGM, compared to 14.8 % of those who are partially literate, and 34.2 % of those who are fully literate (not shown).

The relationship between labour market status and attitude towards FGM is considerably weaker. The lowest support for the discontinuation of FGM exists among women in agriculture, either self-employed (5.6 %), or employees (5.3 %), while the strongest support for the discontinuation was found among service workers (34.8 %), clerical workers (37.7 %), and among women in professional, technical or managerial occupations (45.4 %). Of the non-working women 20.8 % favoured the discontinuation of FGM. The effect of household wealth on attitudes towards FGM was found to be curvilinear, with both the lowest (score = 0) and the highest scores (score ≥ 6) being most likely to favour the discontinuation of FGM (not shown).

Table 2 shows the results of the multilevel logistic regression analyses for whether women favour the discontinuation of FGM or not. In the first model only the individual level variables were included and a random intercept was assumed. Opposition to FGM is most common in the urban and frontier governorates, and significantly less in the rest of Egypt, and especially in the rural areas. Contrary to expectations, after controls for the other variables in the model the likelihood to oppose FGM increases with age. The odds ratio for favouring the discontinuation of FGM comparing a 40 year old respondent with a 20 year old one who score the same on all other variables is 1.58 (95 % CI: 1.30–1.93).

**Table 2:** Multilevel logistic regression results for opposition to the continuation of FGM in Egypt

b (95 % CI)	(1)	(2)
Constant	−0.139	−0.754***
	(−0.776–0.498)	(−1.140–−0.368)
Region (ref: Urban Governorates)	***	***
Lower-Egypt - Urban	−0.324***	−0.331***
	(−0.498–−0.150)	(−0.511–−0.151)
Lower-Egypt - Rural	−0.919***	−0.937***
	(−1.274–−0.564)	(−1.309–−0.565)
Upper-Egypt - Urban	−0.537***	−0.548***
	(−0.657–−0.417)	(−0.662–−0.434)
Upper-Egypt - Rural	−1.018***	−1.037***
	(−1.208–−0.828)	(−1.251–−0.823)
Frontier Governorates	−0.116	−0.120
	(−0.339–0.107)	(−0.349–0.109)
Religion (ref: Muslim)	***	***
Christian	1.623***	1.665***
	(1.396–1.850)	(1.432–1.898)
Other/missing	−0.132	0.280
	(−0.350–0.086)	(−0.002–0.562)
Respondent circumcised	−2.150***	−2.202***
	(−2.446–−1.854)	(−2.520–−1.884)
Number of children born	−0.127***	−0.130***
	(−0.141–−0.113)	(−0.146–−0.114)
Age	0.023***	0.024***
	(0.013–0.033)	(0.014–0.034)
Rural residence	0.076	0.078
	(−0.200–0.352)	(−0.208–0.364)
Labor market position (ref: Not working)	***	***
Professional, technical or managerial occupations	0.157***	0.161***
	(0.096–0.218)	(0.094–0.228)
Clerical	0.243***	0.253***
	(0.127–0.359)	(0.139–0.367)
Sales	−0.132	−0.136
	(−0.344–0.080)	(−0.354–0.082)
Agriculture, self-employed	−0.500***	−0.501***
	(−0.706–−0.294)	(−0.721–−0.281)
Agriculture, employee	−0.543**	−0.540**
	(−0.935–−0.151)	(−0.924–−0.156)
Services	0.256**	0.259**
	(0.093–0.419)	(0.098–0.420)
Skilled manual labor	−0.130	−0.133
	(−0.271–0.011)	(−0.276–0.010)
Other/missing	−0.229	−0.232
	(−0.697–0.239)	(−0.698–0.234)
Education level (ref; No formal education)	***	***
Primary	0.094	0.094
	(−0.010–0.198)	(−0.012–0.200)
Secondary	0.501***	0.508***
	(0.232–0.770)	(0.243–0.773)
Higher	1.138***	1.158***
	(0.777–1.499)	(0.809–1.507)
Literacy (ref: Illiterate)	***	***
Partially literate	0.321***	0.327***
	(0.258–0.384)	(0.260–0.394)
Fully literate	0.552***	0.560***
	(0.311–0.793)	(0.299–0.821)
Basic amenities	0.036**	0.037**
	(0.012–0.060)	(0.010–0.064)
EDHS wave (ref: 1995)		***
2000		0.029
		(−0.251–0.309)
2003		0.190
		(−0.086–0.466)
2005		0.691***
		(0.658–0.724)
2008		0.952***
		(0.915–0.989)
2014		1.010***
		(0.947–1.073)
var(Constante)	0.100*	0.000
	(0.016–0.184)	(0.000–0.000)

Legend: b: partial logistic regression coefficient; 95 % CI: 95 % confidence interval for coefficient

Significance: ***: *p* < .001, **: *p* < .010; *: *p* < 0.050

That tradition is an important factor affecting attitudes towards FGM is demonstrated by the fact that all three tradition variables have strong effects on the likelihood that women favour the discontinuation of FGM. Whether a woman is circumcised or not is very important: the odds to favour the discontinuation for non-circumcised women is 8.58 times (95 % CI: 6.39–11.54) that of circumcised women. Religion proves to be another important factor. As most respondents with a missing value for religious affiliation (mostly those interviewed in 2000 and 2003) are Muslim it is not surprising that this group does not differ significantly from the Muslims. Christians, however, are much more likely than Muslims to oppose FGM (OR = 5.07, 95 % CI: 4.04–6.36). Women with more children, *ceteris paribus*, are also less likely to oppose FGM. For instance, the odds of opposing FGM for a woman with 5 children are only 0.53 times (95 % CI: 0.49–0.057) that of a woman without children.

The effects of women’s social position variables are consistent with those described in the bivariate section, even after controlling for the other variables in the model. The higher the education level of a woman, the more likely she is to oppose FGM. For instance, for respondents with higher education the odds to oppose FGM are 3.12 times greater (95 % CI: 2.18–4.48) than among those without any formal education. Similarly, literate women also are more likely than illiterate women to oppose FGM. Women employed in professional, technical or managerial occupations or in clerical or service jobs are also more likely to oppose FGM than non–working women, while those working in agriculture are less likely to oppose FGM. Women living in wealthier households, as indicated by the basic amenities index, are also more likely to oppose FGM. The odds of opposing FGM for a woman who scores the maximum (=8) on the basic amenities index are 1.33 times higher (95 % CI: 1.10–1.61) than for a woman scoring the minimum (=0). These results confirm that the effects of women’s social position indicators are robust when controlling not only for the individual level control variables, but for the other indicators of a woman’s social position as well.

The second model adds the EDHS wave explicitly to the equation. This allows us to test for overall changes in the attitudes towards FGM, irrespective of the characteristics of the women. The results indicate that after controlling for other actors no significant overall changes are observed between 1995 and 2003. This implies that increases in the opposition to FGM during this period are largely due to changes in the composition of the population (improvements in education, etc.). However, from 2005 onward a significant change in the overall attitude towards the continuation of FGM occurs: compared to 1995, women in the 2005 EDHS have an OR of 2.00 (95 % CI: 1.93–2.06) for favouring the discontinuation of FGM, in 2008 an OR of 2.59 (95 % CI: 2.50–2.69), and an OR of 2.75 (95 % CI: 2.58–2.92) in 2014.

**Fig. 3:**
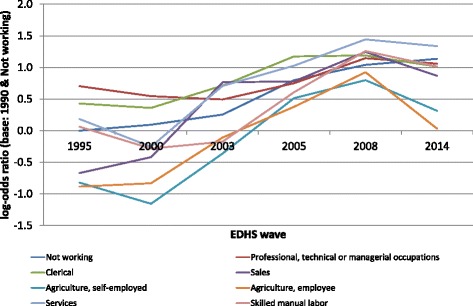
Graphic representation of the interaction effects of labour market position with EDHS wave

**Fig. 4:**
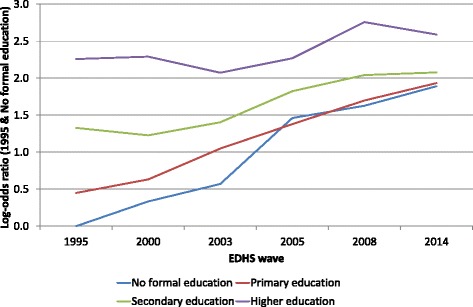
Graphic representation of the interaction effects of education with EDHS wave

A final series of models included the interaction terms between labour market position and education on the one hand and the EDHS survey wave on the other. These models allow us to check how the effects of these women’s position indicators vary over time. For literacy and the basic assets and amenities index no significant random slopes were observed, and therefore no interaction with EDHS wave was tested. The results are presented in Figs. 3 and 4. In these figures the baseline is formed by the reference category of the woman’s position indicator, i.e., not working for labour market position and no formal education for education, in the 1995 EDHS. Figure 3 shows the effects of labour market position across the EDHS waves. All interaction terms are significant at p < 0.050 except those for being self-employed in agriculture, being an agricultural worker in 2000 and 2014, having a clerical job in 2003 or a service job in 2005. This implies that for these specific years the effects of these categories do not differ from their effects in 1995. The results show a clear convergence between the various labour market position categories over time. The categories that had the largest positive effects in 1995, i.e., respondents in professional, technical or managerial occupations and those in clerical occupations, tended to have negative interaction terms in the subsequent EDHS waves. By contrast, those with the most negative main effects, i.e., respondents working in sales or in agriculture, tended to have positive interaction terms in the subsequent EDHS waves. The two extremes therefore are moving toward each other with regard to their attitudes towards FGM, at least up to 2008. For instance, where the odds-ratio for favouring the discontinuation of FGM between respondents in professional, technical or managerial occupations and agricultural workers in 1995 was OR = 4.89 (95 % CI: 3.22–7.43; p < 0.001), by 2008 this OR had become non-significant (OR = 1.25; 95 % CI: 0.90–1.73), but by 2014 it had increased again to OR = 2.79 (95 % CI: 2.21–3.54, p < 0.001). A convergence can also be observed for the effects of education (see Fig. 4). As before, all interaction terms are significant (p < 0.001) except for primary education in 2003. All the interaction effects indicate that the effects of the various education levels (compared to the respondents without formal education) on opposition to FGM decreased over time. For instance, in 1995 the odds of opposing FGM was 9.57 times higher for respondents with higher education than for those without formal education (95 % CI: 7.01–13.07). By 2014 this odds ratio had declined to 2.01 (95 % CI: 1.57–2.59).

## Discussion and conclusions

The results discussed above support earlier findings that female opposition to FGM is most pronounced among well-educated women [5, 6, 9, 33, 43, 45, 46, 53]. The proportion of ever-married Egyptian women who favour the discontinuation of FGM increases with their level of education as well as with employment in the more modern sectors. It is the better educated and less traditional groups that form the vanguard of the anti-FGM movement in Egypt. Resistance to attempts to eradicate and control FGM stems from its embeddedness in traditions and community structures, and its link to the status of women and their families.

Female empowerment strategies often prove effective because they also focus on improving women’s education and literacy. As Jejeebhoy [44] already mentioned, education improves a woman’s position through multiple mechanisms. It not only enhances the economic and social autonomy of women, thus providing alternative paths to status, but it also improves their psychological and cultural outlook. Education not only provides additional knowledge, but also carries what is labelled as ‘modernity’. This involves new social, economic and political institutions, but also a new way of thinking. Modernity entails rationalization and reflexivity, the idea that society is makeable and that people control their own fate [62, 63]. This modern reflexivity allows people to challenge traditional views and practices, and helps them look beyond what they are accustomed to. The changes in knowledge and attitudes about FGM should be seen not as isolated elements but as manifestations of wider cultural changes affecting more domains of life. These cultural changes challenge traditional views of gender roles and relations, which not only refer to the position of women in society, but also to the role and importance of the extended family and community.

Our evidence shows that in Egypt the empowerment of women only is a partial success. During the past few decades a tremendous increase in the education levels of women occurred, but female labour force participation remained dismal. Egypt continues to have a high degree of gender inequality and generally speaking the social position of women remains weak.

Nevertheless, the opposition to FGM among ever-married women increased substantially over the period studied, 1995–2014. Improvements in education and the diffusion of new attitudes may be sufficient for a wider attitudinal change. The data suggest that the initial increase in opposition to FGM occurred mainly because of the growth of those segments of society most likely to oppose FGM, particularly due to the increase in the number of educated women. However, from 2003 onward, opposition to FGM started to spread from the better educated and less traditional segments of society to almost all segments of Egyptian society. This implies that the effects of important predictors, such as education and labour force participation, decreased over time, as the lower educated groups start to catch up with the better educated ones, and the differences among the various socio-economic groups declined.

However, this large scale attitudinal shift may be insufficient to bring FGM to a halt, as the women are usually not in a position to make decisions about FGM. Decisions about whether or not to have a daughter cut are not made by the mother in isolation, but by the larger family. The family is likely to take into account the views of the community as FGM reflects on the status of both the family and the girl herself. Individual mothers have too little power in these networks to block the decision made, but as a group women do play a crucial role as guardians of tradition. Being cut is seen as one’s ticket to becoming a member in good standing of the women’s community [64]. Nevertheless, individual attitudinal changes are an import link in the process of the eradication of FGM. To end FGM, it may not be sufficient that individual women oppose FGM; it may be necessary that entire families and communities adopt anti-FGM views. Although the media can play a role in disseminating anti-FGM messages, the diffusion of anti-FGM attitudes still largely occurs through social networks, although not necessarily through direct contacts. Opposition to FGM has to grow one person at a time until a critical mass is reached within a family, a community, or the country [51, 55, 65]. Behaviour change will become more likely when people are not only ready to change, but willing and able as well. Such interpretation is consistent with social convention theory. Social conventions require social support. To change social conventions, role models (such as individuals or families in good social standing) may introduce new behaviours [55].

Anti-FGM legislation can also contribute to an attitudinal change toward FGM and may help eradicate the practice. The increased opposition to FGM in ever larger parts of Egyptian society open prospects for the legal measures against it. In the past legal measures were ineffective because they had little legitimacy among the population and therefore were rarely enforced. Now that public opinion is changing against FGM existing legislation may become more effective, although one should not expect the practice to stop all at once. The practice of FGM cannot be eradicated until it has become delegitimized in much of Egyptian society. Currently FGM still enjoys too much support of the population, especially outside the major cities, for the legislation to be a success. The importance of the anti-FGM-legislation may be less in its immediate effect in banning the practice, than in its longer term contribution to its delegitimation. Although the legislative action in 2007 and 2008 seemed a step in the right direction, the social and political upheaval from 2011 onward may have halted opposition to FGM. There are several reports that the Muslim Brotherhood and the Mosni government did not merely silently condone the practice but actually promoted it [66–69].

This study has a number of limitations. First, this study makes use of the EDHS which contains only ever-married women. Because younger single women may be more likely to oppose FGM, the DHS data may underestimate the increase in anti-FGM attitudes [6]. As the sample does not contain any never-married women it most likely is not fully representative for the younger, better educated women who may be in the vanguard of the anti-FGM movement. Second, this study only looks at the attitudes of women towards FGM (whether they favour its discontinuation), but does not look at whether they actually have their daughters cut. The literature shows that there are substantial discrepancies between attitudes, intentions and actual behaviour. Many of the women who oppose FGM may still have their daughters cut as they face substantial social pressure to do so. Thus, while the changes in attitudes may be a first step towards the eradication of FGM, the eradication of FGM may still be a long way off. Social norm change will be required and there may need to be a critical mass of women who are educated, uncut, and employed in independent income-generating activities.

## Abbreviations

CI: Confidence interval
DHSs: Demographic and Health Surveys
EDHS: Egypt Demographic and Health Survey
FGM: Female Genital Mutilation
IRB: Institutional Review Board
OR: Odds-ratio
SD: Standard deviation
UNDP: United Nations Development Programme
WHO: World Health Organization

## Footnotes

1 The standard DHS wealth index is calculated for each survey wave separately and is not suitable for comparisons 1 over time.
